# Managing the immune microenvironment of osteosarcoma: the outlook for osteosarcoma treatment

**DOI:** 10.1038/s41413-023-00246-z

**Published:** 2023-02-27

**Authors:** Hailong Tian, Jiangjun Cao, Bowen Li, Edouard C. Nice, Haijiao Mao, Yi Zhang, Canhua Huang

**Affiliations:** 1 State Key Laboratory of Biotherapy and Cancer Center, West China Hospital, West China School of Basic Medical Sciences & Forensic Medicine, Sichuan University, and Collaborative Innovation Center for Biotherapy, Chengdu, 610041 China; 2 Department of Biochemistry and Molecular Biology, Monash University, Clayton, VIC 3800 Australia; 3 Department of Orthopaedic Surgery, The Affiliated Hospital of Medical School, Ningbo University, Ningbo, Zhejiang 315020 People’s Republic of China; 4 Department of Orthopaedics, The First Affiliated Hospital of Zhengzhou University, Zhengzhou, 450052 China

**Keywords:** Bone cancer, Cancer

## Abstract

Osteosarcoma, with poor survival after metastasis, is considered the most common primary bone cancer in adolescents. Notwithstanding the efforts of researchers, its five-year survival rate has only shown limited improvement, suggesting that existing therapeutic strategies are insufficient to meet clinical needs. Notably, immunotherapy has shown certain advantages over traditional tumor treatments in inhibiting metastasis. Therefore, managing the immune microenvironment in osteosarcoma can provide novel and valuable insight into the multifaceted mechanisms underlying the heterogeneity and progression of the disease. Additionally, given the advances in nanomedicine, there exist many advanced nanoplatforms for enhanced osteosarcoma immunotherapy with satisfactory physiochemical characteristics. Here, we review the classification, characteristics, and functions of the key components of the immune microenvironment in osteosarcoma. This review also emphasizes the application, progress, and prospects of osteosarcoma immunotherapy and discusses several nanomedicine-based options to enhance the efficiency of osteosarcoma treatment. Furthermore, we examine the disadvantages of standard treatments and present future perspectives for osteosarcoma immunotherapy.

## Introduction

Osteosarcoma ranks first among malignant bone-related cancers in adolescents and has a complex heterogeneity and an abnormally produced immature osteoid matrix.^1–3^ Currently, the standard treatments for osteosarcoma are neoadjuvant chemotherapy (presurgery), surgical resection, and adjuvant chemotherapy (postsurgery).^4,5^ Despite the efforts of researchers, there has been no significant improvement in the 5-year survival rate of osteosarcoma patients over the past few decades, suggesting that existing therapeutic strategies are insufficient.^6–8^ Moreover, the above approaches cannot effectively eliminate all osteosarcoma cells due to nonspecific drug delivery, which is especially true for metastatic and circulating osteosarcoma cells, which might promote tumor recurrence and progression.^9,10^ Consequently, new therapeutic strategies against osteosarcoma urgently need to be explored.

**Fig. 1:**
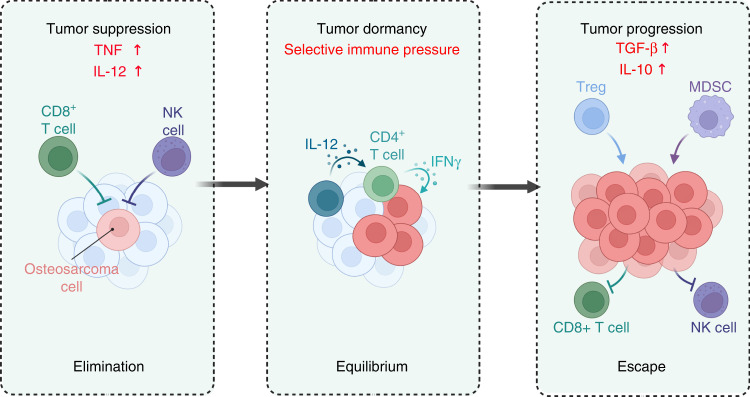
The three stages of immune system attack of tumors: immune clearance, balance and escape

Recently, evidence has shown that the body’s immune system may be in a constant battle with osteosarcoma cells including three stages: immune clearance, balance, and escape^11–13^ (Fig. 1). Moreover, the immune system plays an important role in the execution and exertion of antitumor immunity. Therefore, utilizing the immunity of the organism for more efficient suppression and treatment of cancer has become a focus for researchers.^11,14^ The concept of harnessing the immune system for this purpose originated over 100 years ago when a physician named William Coley successfully treated several of his cancer patients with a combination of live and attenuated bacteria, later known as “Coley’s toxins”, after observing a subset of prior patients enter remission following their diagnosis with the common bacterial infection erysipelas.^15,16^ Notably, the immune microenvironment, the dictator of osteosarcoma treatment response, facilitates cancer cell escape of immune surveillance.^17,18^ Therefore, therapeutic agents that modulate the immune microenvironment and use existing immunity to eliminate osteosarcoma cells are gradually being recognized as new options with great application prospects.^19,20^ Unsurprisingly, immunotherapy shows benefits in terms of potent anti-osteosarcoma effects and suppression of metastasis and recurrence in comparison with conventional intervention strategies, including surgical resection and chemotherapy, which also show satisfactory efficacy in suppressing advanced osteosarcoma.^21–23^

Along with rapid advances in immunology and biotechnology, nanoparticles have shown great promise for enhancing cancer immunotherapy.^24–26^ On the one hand, nanoparticles can effectively improve the pharmacokinetic parameters and reduce the side effects of therapeutic or imaging agents in cancer treatment by site-specific drug delivery.^27,28^ On the other hand, they can also target immune cells and organs to modulate the immune microenvironment to augment tumor immunotherapy.^29,30^ Therefore, versatile nanoplatforms, including biomimetic nanoparticles, inorganic nanomaterials, and organic nanomaterials, have been used to effectively modulate the immune microenvironment.^31–33^ Importantly, some immunomodulatory-based nanoplatforms have achieved satisfactory therapeutic effects in the preclinical study of osteosarcoma.^34^

For these reasons, managing the osteosarcoma immune microenvironment and using nanomedicines in enhanced immunotherapy are gaining widespread attention as personalized treatment regimens. This review, therefore, focuses on the current understanding of the characteristics and functions of the main immune components in the tumor microenvironment, including dendritic cells, T lymphocytes, tumor-associated macrophages (TAMs), tumor-associated neutrophils (TANs), and natural killer (NK) cells. Moreover, we highlight that the benefits of nanomedicines in activating immune responses and reversing the immunosuppressive microenvironment hold great potential in osteosarcoma immunotherapy. Furthermore, the current challenges and future prospects of osteosarcoma immunotherapy are also discussed.

## The immune microenvironment of osteosarcoma

**Fig. 2:**
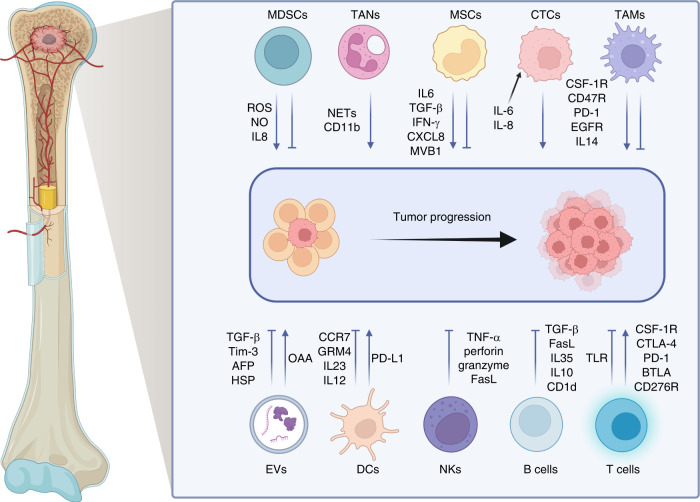
The components in the immune microenvironment of osteosarcoma and the mechanisms of their protumor and antitumor effects

Osteosarcoma tissue is surrounded by massive immune cell infiltration, resulting in the creation of a complex immune microenvironment that allows osteosarcoma cells to grow within the bone by creating an immunosuppressive microenvironment to maintain their survival and proliferation.^35–37^ A robust immunosuppressive microenvironment is positively correlated with overactivation of molecules associated with immune suppression, such as indoleamine 2,3-dioxygenase (IDO), programmed cell death protein 1 (PD-1), interleukin-10 (IL-10), transforming growth factor-β (TGF-β), vascular endothelial growth factor (VEGF), and signal transducer and activator of transcription 3 (STAT3), due to their immunosuppressive effects mediated by myeloid-derived suppressor cells (MDSCs), TAMs, and regulatory T lymphocytes (Tregs)^38–43^ (Fig. 2). Consequently, there is an urgent need to gain an in depth understanding of and characterize the osteosarcoma immune microenvironment to develop advanced immunotherapies by utilizing these immunologic biomarkers.

### Dendritic cells (DCs) in the osteosarcoma immune microenvironment

DCs, the most common antigen-presenting cells (APCs) originating from the bone marrow, are mainly divided into DCs, DC1s, and DC2s.^44–47^ It should be noted that type 1 myeloid/conventional DCs (cDC1s) have an excellent profile of antigen presentation and cross-presentation and efficient T lymphocyte priming activity for initiating the immune response.^48–50^ There are significant differences in inflammatory infiltration among various types of osteosarcoma, but there is no difference in DCs. For instance, (DC-SIGN/CD11c^+^) DCs are more common in conventional high-grade osteosarcoma than in other sarcomas.^51^ Moreover, DC infiltration has been found to be associated with autophagy in osteosarcoma. For example, a recent study using a machine learning-based autophagy-related long noncoding RNA signature showed the association between infiltration of immune cells and the expression of autophagy-related genes, among which RUSC1-AS1 was adversely connected to the numbers of infiltrating immature DCs, mast cells, and macrophages.^52^ With osteosarcoma progression, osteosarcoma cells can develop DC- and phagocytosis-tolerant variants, which reduces DC activation and ultimately causes immune escape.^53^ It is well known that DCs can express glutamate metabotropic receptor 4 (GMR-4) and carcinogens to drive osteosarcoma pathogenesis. Agonists of GMR-4 or antibodies against IL-23 may be potential options for osteosarcoma immunotherapy.^54,55^ Additionally, DCs may also play a significant role in the pulmonary metastasis of advanced osteosarcoma.^56^ A comprehensive study showed that CCR7 contributed to the proliferation, deformation, and migration of DCs, thereby playing an important role in pulmonary metastasis of osteosarcoma. This work also suggested that the number of CD1c^+^ DCs was higher in pulmonary metastases than in primary and recurrent lesions.^57^

Several lines of evidence support the potential therapeutic value of DCs in osteosarcoma, but there are also some contradictory views. For example, PD-L1 levels were strongly related to the quantity of DCs and T lymphocytes in osteosarcoma.^58^ Additionally, DCs and TAMs were also found to be closely associated with survival time. Moreover, some studies have also suggested links between DCs and osteosarcoma prognosis based on immune classification, with fewer DCs than cytotoxic T lymphocytes and NK cells found in living patients.^59^

### T lymphocytes in the immune microenvironment of osteosarcoma

T lymphocytes, a type of thymus-derived lymphocyte, mature and reside in peripheral immune organs.^60,61^ They contribute to cellular and humoral immunity and can be classified according to different criteria. For example, they are usually classified into effector, naive, and memory T lymphocytes during the activation stage.^62,63^ Based on the features of cell receptors, such as major histocompatibility complex (MHC) restriction and biodistribution, these cells are divided into αβT and γδT lymphocytes. In addition, they can also be divided into cytotoxic T lymphocytes (CTLs), helper T lymphocytes (Th lymphocytes), and Tregs according to their function.^64–66^ Overall, T lymphocyte classification is highly heterogeneous, and T lymphocyte infiltration plays a significant role in osteosarcoma immunotherapy.

**Fig. 3:**
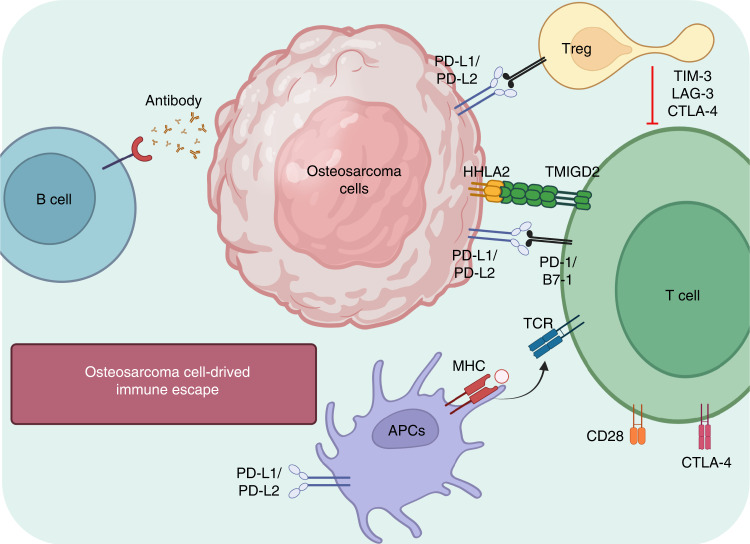
Schematic illustration of immune checkpoints and the interplay between immune cells and osteosarcoma cells

The majority of tumor-infiltrating lymphocytes are clustered at regions overexpressing human leukocyte antigen (HLA) class I in osteosarcomas, while effector T lymphocytes are mostly distributed at the border between healthy tissues and pulmonary metastases.^67,68^ Moreover, there are more T lymphocytes in metastases than in primary or recurrent lesions in situ.^69^ However, the levels of immune checkpoint and immunomodulatory molecules in metastatic lesions, such as PD-1, IDO, IFN-γ, and T lymphocyte immunoglobulin and mucin domain-containing protein-3 (TIM-3), have been found to be higher than those in primary tumors^68,70–72^ (Fig. 3). A recent report investigated infiltrating T lymphocytes in biopsy tumor tissue and peripheral blood samples from sixteen primary osteosarcoma patients,^73^ showing that there were more TIM-3^+^ PD-1-negative or -positive T lymphocytes in tumor tissue than in blood circulation, which indicated that the osteosarcoma immune microenvironment was suppressive. This study also showed that these immune infiltrating cells could promote the formation of an immunosuppressive microenvironment via crosstalk with each other in osteosarcoma, which suggested that the immune function of T lymphocytes could be suppressed by M2-type TAMs and that the consumption of CD163^+^ M2-type macrophages could activate the function and proliferation of T lymphocytes and the secretion of proinflammatory factors by M1-type macrophages.^74^ In conclusion, complex T-lymphocyte infiltration occurs in different regions and subgroups of osteosarcoma, involves various molecules, and plays different roles in antitumor immune responses.

### Natural killer cells (NKs) in the immune microenvironment of osteosarcoma

NKs, considered nonspecific cytotoxic immune cells, are able to nonspecifically destroy infected abnormal cells (such as cancer cells) without prior activation or sensitization.^75–77^ They generally express suppressive surface receptors, such as killer-cell immunoglobulin-like receptors (KIRs), that can identify specific HLA class I molecules, including CD94/NK group 2 member A (NKG2A) and HLA-A, B and C.^78–80^ Moreover, they can be trained to lyse cancer cells with low expression of MHC class I produced from host cells.^81,82^ Their activated surface receptors generally include NKG2D and natural cytotoxic receptors (NCRs), which can recognize stress proteins on the surface of cancer cells, such as MHC class I peptide A/B (MICA/B), UL16-binding proteins (ULBPs), and the Fcg receptor CD16, which induces ADCC by recognizing the Fc portion of antibodies on opsonized cells.^83–86^ Coreceptors of NKG2D and NCRs, such as DNAM-1, can enhance the activation of NK cells for efficient immune responses.^87^ The balance between positive and negative signals received by NK cells determines their antitumor effects, which are mainly modulated by the secretion of cytotoxic granules (such as perforin and granzyme), the generation of cytokines (such as IFN-γ and TNF-α) activating antitumor immunity, and the expression of death receptor ligands on their surface.^88^

Osteosarcoma cells have been shown to be readily eliminated by NK cells in some preclinical studies.^89,90^ For example, cultivation of NKs from normal donors with osteosarcoma feeder cells for one week resulted in a median killing effect of approximately 46.1%.^91,92^ Notably, this cytotoxicity was not associated with the expression levels of NK receptor ligands but was significantly suppressed through the exposure of NKs to anti-DNAM-1 and anti-NKG2D antibodies.^93^ Similarly, blockade of the NKG2D receptor, but not that of DNAM-1, could greatly reverse the cytotoxicity of NK cells against osteosarcoma cells in in vitro assays.^94,95^ KIRs also play a significant role in osteosarcoma treatment: KIR receptor-ligand mismatched NK cells have an excellent in vitro anti-osteosarcoma effect, and this effect is further augmented when the HLA class I molecule is blocked in osteosarcoma cells.^94,96,97^ Furthermore, intraperitoneal administration of IL-2 in combination with intratumoral administration of activated and expanded NK cells can effectively mitigate bone impairment, suppress osteosarcoma volume, inhibit pulmonary metastasis, and clearly prolong mouse survival time.

### The tumor-associated macrophage (TAM)-mediated immunosuppressive microenvironment in osteosarcoma

TAMs are generally considered to be derived from the myelomonocytic lineage and to develop from hematopoietic stem cells (HSCs).^98–100^ Moreover, they are generally recruited from the blood circulation to the site of the lesion to eliminate infection, inflammation or tumor cells, such as osteosarcoma cells.^101–103^ However, emerging evidence has recently indicated that TAMs can develop in embryos before the emergence of HSCs and maintain self-renewal and proliferation.^104,105^ Macrophages can be divided based on their origin into tissue-resident macrophages, mainly derived from the yolk sac, and blood monocytes, derived from the fetal liver and bone marrow.^106^

The quantity of TAMs may vary markedly in different solid tumors, including osteosarcoma, but they are the most abundant immune cell type in the tumor microenvironment, accounting for nearly 50% of the total tumor cells.^38,107^ TAMs play a significant role in matrix remodeling, inflammation and vascularization in antitumor immunity and modulation. In general, TAMs are characterized by protumor or antitumor effects based on the degree of malignancy of the tumor and their interactions with the tumor microenvironment because of their plasticity and heterogeneity.^108,109^ For instance, type-1 TAMs have the capability to phagocytose cancer cells and promote the secretion of inflammatory factors to improve antitumor immune responses.^110,111^ However, TAMs usually show an immunosuppressive type-2 phenotype in the tumor microenvironment and are prone to facilitate angiogenesis and extravascular invasion, which promote evasion of immune surveillance, eventually resulting in tumor progression, metastasis, and relapse.^112,113^ Moreover, CD14 and CD68 double-positive TAMs are the main immune infiltrating TAM subtype in bone-associated cancers, including osteosarcoma.^51^ An analysis of osteosarcoma patient RNA expression profiles, clinical features, and immune cell proportions showed that type 2 TAMs are the main immune cell type and are closely related to survival time.^114^ Another study using CD209 staining and gene expression analysis supported that there is accumulation of type-2-like TAMs in human osteosarcoma tissues and found that retinoic acid could modulate M2-like TAMs to suppress osteosarcoma initiation and stemness.^115^

Recently, emerging evidence has also confirmed that the quantity of TAMs in various tumor tissues is closely associated with the quantity of tumor blood vessels, suggesting that TAMs promote tumor angiogenesis.^116–118^ For example, various proangiogenic substances, such as fibroblast growth factor (FGF), matrix metallopeptidase 9 (MMP-9), and vascular endothelial growth factor (VEGF), are produced by TAMs to promote tumor progression and metastasis in various cancers, including osteosarcoma.^119–121^ Moreover, TAMs can also interact with various immune effector cells to induce an immunosuppressive tumor microenvironment. They can suppress the activity of T lymphocytes to facilitate tumor immune escape by overexpressing PD-1 and cytotoxic T lymphocyte-associated antigen-4 (CTLA-4) receptors.^122^ Furthermore, M2-like TAMs can also increase vascular extravasation, promote the survival and growth of metastases, suppress cytotoxic T lymphocytes, and maintain immunosuppressive Tregs to enhance tumor invasion and metastasis.^123^ As a result, the premetastatic niche is formed at distant lesions in specific metastatic sites, including bone, lung, and liver, with the assistance of TAMs.^124^

In addition to the above findings, recent reports have also shown that TAMs participate in local inflammatory regulation and drug resistance in osteosarcoma by interacting with other immune cells in the tumor microenvironment.^125^ However, distinct TAM subtypes may respond differently in osteosarcoma, causing different degrees of malignancy. Specific targeting of TAMs (such as CD163-positive TAMs) rather than pandepletion of TAMs has been shown to enhance the cytotoxic activity of T lymphocytes functioning in tumor suppression.^73,126,127^ Such information might prompt researchers to define specific TAM features and subtypes in human biopsies for enhanced TAM-specific targeting. Indeed, specific TAM subgroups, characteristics and signals continuously evolve over immunological progression, modulating either protumor or antitumor activity (summarized in Table 1). We have also highlighted the differences between human and murine TAMs (summarized in Table 2). Overall, with the deepening understanding of TAMs, investigators will have the option to manipulate TAMs using various approaches to enhance osteosarcoma immunotherapy.

**Table 1:** Therapeutic TAM-modulating agents for osteosarcoma

Therapeutic agent	Targeted cell or molecule	Mechanism	Reference
Mifamurtide (MTP-PE)	Monocytes and macrophages	Switches TAM polarization toward an intermediate M1-M2 phenotype	^474^
	pSTAT3, pAKT, IL-17R TNF-a, IL-1, IL-6, IL-8, NO, PGE2, and PGD2 LFA-1, ICAM-1 and, HLA-DR	Switches TAM polarization toward an intermediate M1-M2 phenotype	^475^
ATRA	CD117^+^ Stro-1^+^ cells, cancer stem cells, and macrophages	Decreases M2-like TAMs	^125^
	IL-1b, IL-4, IL-6, IL-13, and CXCL8	Decreases M2 phenotype polarization-mediated stemness	^476^
Esculetin	LM8 cells and macrophages Cyclin D1, CDK 4, MMP-2, TGF-b1, VEGF, IL-10, MCP-1, and pSTAT3	Downregulates essential cytokines (TGFb1, IL-10, and MCP-1) and proteins (pSTAT3) Involved in the differentiation of M2 macrophages	^299^
Zoledronic acid	Monocytes, DCs, and macrophages	Upregulates M1-like cytokines	^73^
	IL-1β, TNF-α, VEGF, IL-10, IDO, IL-12, and poly I:C TGF-β, Arg-1, and Fizz-1	Downregulates M2-like cytokines	^477^
Porous hollow iron nanoparticles	Macrophages PI3K g and NF-kB p65	Upregulates NF-kB p65 and downregulates PI3K g in TAMs	^478^
Chimeric antigen receptor macrophages	T cells, dendritic cells, and macrophages ERK and NF-kB (P65)	Upregulates pro-inflammatory pathways (interferon signaling, the TH1 pathway, and iNOS signaling) in M2 macrophages	^479^

**Table 2:** Differences between mouse and human macrophages

Parameters	Murine TAMs	Human TAMs	Therapeutic implications
M1-type versus M2- type polarization factors	M1: IFNγ+LPS M2: M-CSF+IL-4	M1-type: GM-CSF, GMCSF+IFNγ, CSF+IFNγ M2: M-CSF+IL-4 (or IL-1 or IL-10)	The M1/M2 model is not relevant for human TAM characterization
Specific markers	M1: iNOS, CD80, MHC-II, IFNγ M2: Arg1, Ym1, VEGF	M1: IL-1β, IL-6, TNFα, IL-12 M2: TGF-β, VEGF, EGF, PDGF, IL-10	Prototypic mouse M1 and M2 markers cannot be used to characterize human TAMs
Type II IFN expression	High expression	Low expression	Type II IFN production by murine TAMs may favor cytotoxic T lymphocyte responses and their reprogramming into antitumor Mφ
Polarization versus activation	Resting (M2) and LPS-stimulated (M1) cells are usually compared	Human Mφ and TAMs require stimulation (such as stimulation via CD40 or TLRs) to reveal their phenotypes	Human Mφ polarization and activation are two independent processes

### The tumor-associated neutrophil (TAN)-mediated immunosuppressive microenvironment in osteosarcoma

Neutrophils are important immune cells that are sensitive to pathogens and tissue impairment, accounting for nearly 50%-70% of total white blood cells in humans.^128–130^ Most clinical studies on neutrophils in osteosarcoma patients have focused on the ratio of neutrophils to lymphocytes or circulating neutrophils, and an increased ratio of neutrophils to lymphocytes pretreatment or presurgery might be closely associated with poor outcomes, indicating that this ratio can be applied as a prognostic marker for osteosarcoma.^131–135^ Neutrophils in the tumor microenvironment, also named TANs, show functional versatility and phenotypic heterogeneity similar to those of TAMs.^136–138^ However, there are limited reports about neutrophil infiltration in the osteosarcoma immune microenvironment.

In osteosarcoma, TANs may have a longer lifespan under the stimulation of proinflammatory factors (such as IFN-γ) than circulating neutrophils.^139,140^ The neutrophil extracellular trap (NET)-mediated immunosuppressive microenvironment plays a significant role in immune escape during cancer immunotherapy and consists of a reticular chromatin structure composed of chromatin and granule proteins produced from neutrophils;^141^ this microenvironment facilitates tumor metastasis instead of conventional phagocytosis and killing factor secretion.^142^ For example, PAD4, which is overexpressed in osteosarcoma, plays an important role in forming NETs via extensive chromatin decondensation.^143^ Moreover, TANs can also be polarized to anticancer N1-like or procancer N2-like neutrophils, which are similar to M1- and M2-like macrophages.^144–146^ In this process, TGF-β produced from tumor and tumor microenvironment-associated cells can effectively promote the aggregation of type-2 neutrophils in tumor regions, resulting in functional and phenotypic neutrophil changes.^144,147^ In addition, the stimulation of TANs with IFN-γ and TNF-α can polarize type-2 neutrophils into type-1 neutrophils to efficiently suppress tumor growth.^148^ This phenotypic switch is closely related to changes in protein secretome profiles, including changes in the levels of secreted granule-associated proteins, adhesion molecules, chemokines, and cytokines.^149–152^ Compared with patients with metastasis, patients with nonmetastatic osteosarcoma present significantly higher expression levels of the neutrophil-specific marker CD11b.^153^ Infiltrating neutrophils further exert positive antitumor effects by coordinating the recruitment of immune cells to effectively mediate specific immunity while activating antibody-dependent cellular cytotoxicity (ADCC).^154,155^ Furthermore, the infiltration of neutrophils has also been reported to be associated with the expression of hypoxia-related genes.^156,157^ Emerging studies have indicated that hypoxia in the tumor microenvironment promotes tumor progression, and thus, hypoxia could be regarded as a prognostic factor for metastasis.^32,158,159^ There are significantly fewer N1-like TANs in groups with high hypoxia than in the groups without hypoxia, suggesting that hypoxia might contribute to evasion of immune surveillance and promotion of tumor metastasis by reducing antitumor immune cells.^157^ However, both studies described above only considered the total TAN number without taking functional differences between subtypes into account, which may be due to the difficulty in recognizing specific biomarkers. Therefore, more comprehensive studies are required to reveal the complex functions of TANs in osteosarcoma; furthermore, advanced treatments related to TANs are still being developed, and novel ideas derived from basic scientific research are required.

### The myeloid-derived suppressor cell (MDSC)-mediated immunosuppressive microenvironment in osteosarcoma

MDSCs, as a group of immunosuppressive immature myeloid cells, are able to create an immunosuppressive tumor microenvironment by differentiating into tumor-associated DCs, TAMs, and TANs.^160,161^ They interact not only with the immune microenvironment but also with osteoblasts, osteoclasts, chondrocytes, and other stromal cells in the bone and joint microenvironment to facilitate the pathogenesis and metastasis of various tumors, including osteosarcoma.^162,163^ Generally, murine MDSCs are characterized by the coexpression of CD11b and Gr-1, and these cells are now further divided into granulocytic (G-MDSCs) and monocytic (M-MDSCs) based on their phenotypes and morphological characteristics. The former express have the cell surface marker phenotype CD11b^+^Ly6ClowLy6G^+^, while the latter are characterized by a CD11b^+^Ly6ChighLy6G^-^ phenotype.^164,165^ Human MDSCs are different from murine MDSCs, which are characterized by no or low expression of HLA-DR and high expression of CD33. Unsurprisingly, the common myeloid biomarker CD11b is also a marker of human MDSCs, and the CD11b and CD33 double-positive HLA-DR-low population can be further classified as M or G MDSCs according to their CD14 or CD15 expression. It should be noted that CD15-positive cells usually exhibit a granulocytic morphology, while CD14-positive cells exhibit monocytic features.^166,167^

Recent reports have confirmed that early bone marrow mesenchymal stem cells (e-BMSCs) can serve as precursors of PMN-MDSCs and M-MDSCs.^162,168,169^ MDSCs interact most closely with T lymphocytes of all immune cells, and these interactions can lead to the production of reactive oxygen species (ROS) and ablate L-arginine in the tumor microenvironment to suppress the proliferation and boost the apoptosis of T lymphocytes and weaken T lymphocyte-mediated immunity.^170,171^ Different MDSC subtypes can also suppress the activity of T lymphocytes in different ways. For example, PMN-MDSCs can upregulate nicotinamide adenine dinucleotide phosphate (NADP) oxidase and activate STAT-3 to generate ROS, while M-MDSCs can modulate inducible nitric oxide synthase and activate STAT1 to release NO to suppress the function of T lymphocytes.^172,173^ Notably, MDSCs can inhibit not only the acquired antitumor immune response but also the innate antitumor immune response.^174,175^ For instance, MDSCs can impair the antigen presentation of DCs and phagocytosis of NK cells to promote tumor immune escape.^176–178^ In addition to suppressing immunity in the tumor microenvironment, MDSCs can also actively participate in the management of the immune microenvironment and tumor metastasis.^179^ Interestingly, under the stimulation of the hypoxic tumor microenvironment, MDSCs usually produce high levels of basic FGF, VEGF, VEGF analog Bv8, and MMP-9 to promote angiogenesis and the creation of a premetastatic niche, indicating a close association with pulmonary metastasis of osteosarcoma.^180^

### Extracellular vesicles (EVs) in the immune microenvironment of osteosarcoma

Cancer cell-derived EVs are a group of heterogeneous nanovesicles secreted into the tumor microenvironment or circulation, encapsulating intact organelles, proteins, nucleic acids, and lipids (such as eicosanes, cholesterol, and fatty acids).^181–183^ Exosomes, a subclass of EVs, are produced by direct outward budding of the cell membrane.^184,185^ They induce the proliferation, metastasis, and chemotherapy resistance of osteosarcoma cells.^185–187^ These functions may be derived in part from interactions between tumor-derived exosomes and bone cells that form a microenvironment conducive to cancer cell homing.^188^ Notably, tumor cell- and immune cell-derived exosomes have been shown to carry TAAs and activate antitumor immune responses, resulting in the elimination of established tumors by CD8^+^ and CD4^+^ T lymphocytes, as well as directly inhibiting tumor cell proliferation and malignant tumor progression.^189^ In addition, emerging evidence supports that tumor-derived EVs can typically form an immunosuppressive microenvironment. As an example, the release of soluble MHC-I chain-related proteins and NKG2D soluble receptors from osteosarcoma-derived exosomes can suppress NK cell or CTL activity, thereby creating a conducive environment for osteosarcoma immune escape.^190,191^ Another study suggested that the level of exosomal PD-L1 in osteosarcoma patients with pulmonary metastasis was much higher than that in patients without pulmonary metastasis.^191^ In the study, a mouse model was used to evaluate the roles of exosomes expressing PD-L1, and the study showed that pulmonary metastasis significantly increased after exposure to such exosomes. Based on these studies, it appears that osteosarcoma cells can secrete exosomal PD-L1 to promote lung metastasis; therefore, detecting the level of exosomal PD-L1 in serum might be an important means to identify pulmonary metastasis for clinical treatment. A potential mechanism behind the utility of this strategy is related to PD-L1-induced immunosuppression, which subsequently enables expression of epithelial-mesenchymal transition (EMT)-related proteins, including N-cadherin, vimentin, fibronectin, and laminin 5. Another study reported that in 36% of cancer patients, exosomes containing IDO, which can modulate antitumor immune responses by regulating tryptophan (Trp) consumption and is involved in the formation of an inflammatory microenvironment to facilitate tumor angiogenesis, were identified in the majority of bodily fluids.^192,193^ These molecules in osteosarcoma-derived exosomes plays an indirect role in immune responses and may influence DC-mediated neoantigen presentation.^190^ Therefore, reversing the immunosuppressive microenvironment by regulating exosome release may be a new strategy to enhance osteosarcoma immunotherapy.

### Mesenchymal stem cells (MSCs) in the immune microenvironment of osteosarcoma

MSCs, considered competitive clones that promote tumorigenesis, are another reason for the substantial heterogeneity found in osteosarcoma, which causes chemotherapy resistance, recurrence, and metastasis.^194^ Based on the expression of tumor stem cell-related genes, osteosarcoma patients can be divided into two clusters (Cluster 1 and Cluster 2).^195^ The immune microenvironment of Cluster 1 patients has fewer follicular helper T lymphocytes and macrophages and more cytotoxic T lymphocytes, resulting in an immune infiltrating phenotype and superior therapeutic effects compared to those of Cluster 2 patients. This suggests that MSCs affect the immune microenvironment of osteosarcoma, and different types of MSCs might lead to diverse immune infiltration phenotypes and outcomes. Moreover, a variety of studies have indicated that osteosarcoma might be derived from bone marrow-derived MSCs.^196–199^ MSCs are mature pluripotent stem cells found in different tissues, particularly in the dental pulp, bone, and adipose tissue, and play a significant role in modulating immunity, cell fusion and differentiation in osteosarcoma tumorigenesis.^200^ The histogenesis of osteosarcoma shows that naive MSCs and MSCs from tumors might play adverse roles in osteosarcoma progression. Naive MSCs generally have suppressive or supportive effects in cancer, while tumor-derived MSCs have the ability to facilitate epithelial-mesenchymal transition (EMT), thus exhibiting a robust immunosuppressive effect and promoting tumor cell proliferation.^201^ This MSC-mediated promotion of the proliferation and metastasis of osteosarcoma cells can be attributed to the following two points. On the one hand, the interaction between MSCs and osteosarcoma cells involves aquaporin 1 and IL-8. On the other hand, abnormal gene expression, including abnormal expression of TP53, Rb, C-MYC, IHH, and KRAS, can facilitate the reprogramming of MSCs into osteosarcoma cells.^199,202–205^ Notably, MSCs can also transform into tumor-associated fibroblasts (CAFs) after exposure to osteosarcoma cells, which can obviously promote osteosarcoma cell proliferation and metastasis.^200,206^ This process usually involves multiple substances, including monocyte chemotactic protein 1, growth-related oncogene-α, TGF-β, and intercellular adhesion factor. Moreover, CAFs can release extracellular matrix components to maintain cell proliferation and intercellular adhesion and communication to maintain malignant phenotypes and increase tumor heterogeneity. To some extent, osteosarcoma cells and MSCs have similar functions. For example, some studies have reported that osteosarcoma cells can also trigger the migration and invasion of endothelial cells and facilitate angiogenesis after exposure to MSCs.^198,206–208^ In regard to the immune response, MSCs can effectively release anti-inflammatory factors and suppress proinflammatory factors to assist osteosarcoma cells in immune escape, which is induced by autocrine or paracrine EVs, especially exosomes.^209^ An interesting study reported that MSCs can secrete EVs containing miRNAs/RNAs and proteins to suppress the proliferation and immune response of T lymphocytes.^210^ Additionally, TGF-β and IFN-γ secreted by MSC-derived EVs can induce the switch of mononuclear cells into Tregs.^211^ In addition to inhibiting T lymphocyte-related antitumor immunity, MSCs can also suppress the immune function of B lymphocytes. For example, MSC-derived exosomes can significantly increase the expression of MVB1 RNA and C-X-C motif chemokine ligand (CXCL) 8, which decreases the quantity of immunoglobulin M (IgM) in B lymphocytes.^212^ It should be noted that MSCs can also secrete IL-6 to promote the M2-like polarization of TAMs.^213,214^ Additionally, some cytokines, including IL10, hepatocyte growth factor (HGF), leukemia inhibitory factor (LIF), CXCL2, CXCL20, and VEGF-C, also play a significant role in the increased accumulation of MSC-EVs at tumor sites and inflammation suppression.^215^

### Circulating tumor cells (CTCs) in the immune microenvironment of osteosarcoma

Cancer cells, including osteosarcoma cells, can exist either in tumor tissues or in blood circulation, and such cells are named circulating tumor cells (CTCs).^10^ They have the ability to escape local therapy to survive at low levels with systemic interventions, such as radiotherapy, phototherapy and surgical resection, eventually leading to the metastasis and recurrence of osteosarcoma.^216,217^ Emerging evidence has also shown the important role of CTCs in the osteosarcoma immune microenvironment.^218^ For instance, previous studies have shown that suppression of IL-6 can decrease the number of CTCs to improve osteosarcoma treatment effects.^219^ This phenomenon was also found in another in vitro study that suggested that human IL-6 could activate the Janus-activated kinase/STAT3 and mitogen-activated protein kinase/extracellular signal modulated kinase 1/2 (MEK-1/ERK) pathways. These pathways have been shown to promote osteosarcoma cell proliferation, but only the Janus-activated kinase/STAT3 pathway can drive the migration of osteosarcoma cells. Therefore, one can speculate that the STAT3 pathway might facilitate the spread of CTCs for the formation of an immunosuppressive osteosarcoma microenvironment. In addition to IL-6, IL-8 also participates in CTC-mediated osteosarcoma progression by recruiting and activating T or B lymphocytes, neutrophils, basophils, and eosinophils. IL-8 released from self-seeded CTCs can effectively induce osteosarcoma cell proliferation and pulmonary metastasis in ex vivo assays.^220^ Consequently, suppressing the activity and secretion of IL-8 might be a potential antitumor strategy that can possibly be used in combination with various agonists or antagonists of other cytokines. Although the mechanism underlying the relationship between immune responses and CTCs is not clear, CTCs have been identified as potential predictive biomarkers and drug intervention targets for enhanced osteosarcoma immunotherapy. Furthermore, elimination of CTCs is being recognized as a more thorough and effective antitumor strategy.

## Conventional immunotherapy for osteosarcoma

**Fig. 4:**
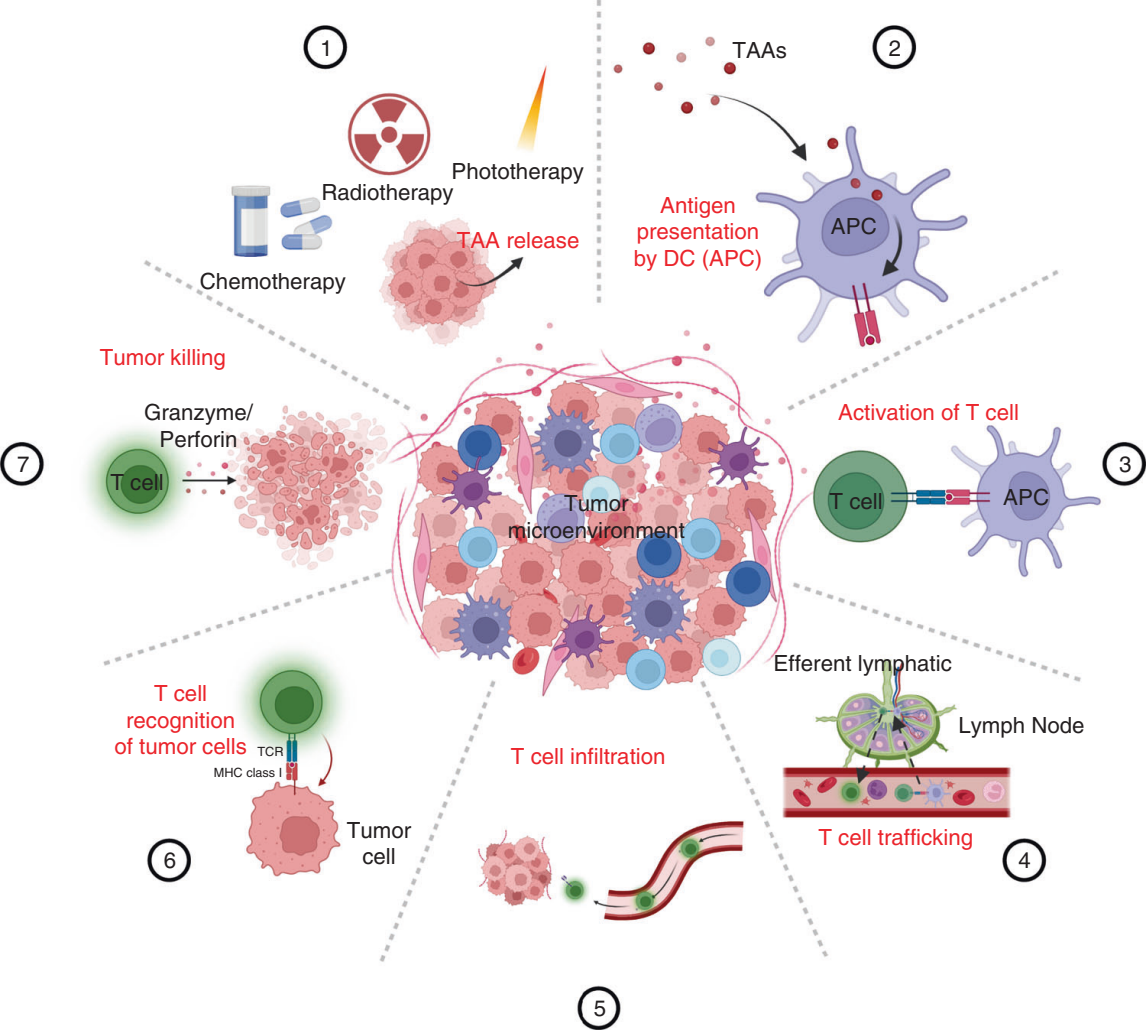
Schematic illustration of the cancer immunity cycle, which can be divided into seven main steps: (1) release of TAA; (2) presentation of TAAs; (3) activation and proliferation of T lymphocytes; (4−5) trafficking and infiltration of T lymphocytes; (6) T lymphocyte recognition of cancer cells; and (7) killing of cancer cells by T lymphocytes

To better understand the immune response in osteosarcoma, the concept of the “tumor immunity cycle” has been used to illustrate approaches to enhance the effects of immunotherapy^221^ (Fig. 4). The crucial process of this cycle occurs in the tumor and regional lymph nodes, with immune cells traveling between these distinct regions.^222–224^ This immunity cycle starts with the release of neoantigens or TAAs from dying cancer cells, and these TAAs are subsequently captured and processed by APCs. After that, APCs present the processed TAAs on MHC-I and MHC-II molecules to naive T lymphocytes in draining lymph nodes, leading to the activation and production of CTLs to eliminate cells with specific TAAs. Activated CTLs multiply through clonal expansion, enter the blood circulation, and migrate from local lymph nodes to the tumor microenvironment. Once activated T lymphocytes arrive at tumor tissues, they can release cytotoxic substances such as perforin and granzyme B to eliminate tumor cells. These dying tumor cells in turn release more TAAs and costimulatory signals (such as DAMPs) to induce the antitumor immunity cascade.^225,226^ However, osteosarcoma can disrupt the essential elements of the tumor immunity cycle via an extensive negative feedback immunoregulatory mechanism, which is becoming increasingly recognized as a promising target for osteosarcoma immunotherapy.^227^

### Macrophage modulation strategies for osteosarcoma immunotherapy

**Fig. 5:**
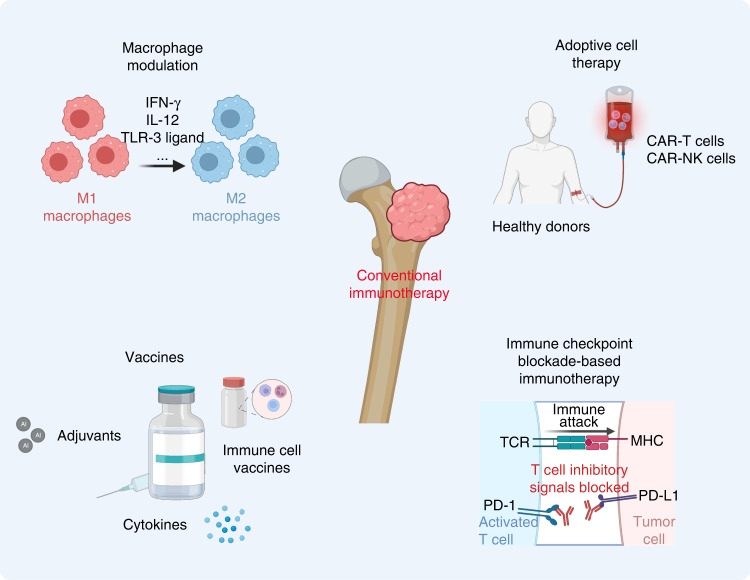
Schematic illustration of current immunotherapy strategies for osteosarcoma

Macrophages are capable of responding to multiple stimuli in the tumor immune microenvironment via broad activation phenotypes due to their plasticity. As mentioned above, the transition from M1- to M2-like polarization of TAMs plays a significant role in the pulmonary metastasis of osteosarcoma. Thus, modulating macrophage polarization has been considered a promising approach for osteosarcoma immunotherapy (Fig. 5). Various agents have been used to modulate the polarization of macrophages to enhance antitumor immune responses, including Toll-like receptor (TLR) agonists, cytokines, and monoclonal antibodies.^228^ Moreover, several cytokines (including IFN-γ and IL-12) have been found to reprogram macrophages toward type 1-like phenotypes by activating the STAT signaling pathway.^229^ It should be noted that TLRs are important pathogen recognition receptors expressed by APCs, such as macrophages; therefore related agonists can mediate the switch of M2- to M1-like phenotypes to elicit an antitumor immune response.^230^ For example, Vidyarthi *et al*. found that murine colonic tumors polarized M2-like TAMs toward M1-like TAMs and inhibited tumor cell proliferation in an IFN-αβ signaling pathway-dependent manner by administering the TLR-3 ligand [poly (I: C)].^231^ In addition to cytokines and TLR agonists, antibodies, such as anti-CSF1 and anti-CD40 antibodies, are also used to facilitate the polarization of TAMs.^232,233^ Moreover, several drugs have also been shown to reprogram TAMs and have showing promising results in osteosarcoma treatment. Lipopolysaccharide (LPS)-activated M1-like TAMs in combination with IFN-γ presented significant inhibitory effects on osteosarcoma cell proliferation, and these effects could be regulated by soluble substances released by TAMs in an IL-1/TNF-α-independent manner.^234^ For example, all-trans retinoic acid (ATRA) can effectively suppress M2-like polarization and the secretion of MMP-12 to inhibit the invasion and pulmonary metastasis of osteosarcoma.^235^ Metformin (Met), which repolarizes TAMs to elicit antiangiogenic and antitumor effects, also plays a significant role in suppressing osteosarcoma cell proliferation by reprogramming the metabolic polarization of TAMs.^236,237^ Notably, gefitinib (Gef), an efficient EGFR inhibitor, can also repolarize the pulmonary macrophage phenotype by disturbing macrophage receptor-interacting protein kinase 2 (RIPK2) expression to suppress the invasion and metastasis of osteosarcoma.^238–240^ Additionally, Gef can also relieve postoperation-accelerated osteosarcoma metastasis and prolong overall survival time in a mouse model of osteosarcoma.^241^ Various compounds have also been derived from natural products, including epimedokoreanin B (derived from Epimedii Herba),^242^ onion A1 (derived from allium sulfides),^243^ and oleanolic acid (OA)/corosolic acid (CA).^244,245^ In an osteosarcoma mouse model, these compounds significant suppressed STAT3 activation to modulate type 2 TAM polarization, protecting against osteosarcoma progression and metastasis. Another study explored M2-like TAM antagonists or modulators, such as esculetin, wogonin (derived from the roots of Scutellaria baicalensis), resveratrol and synthetic hydroxystilbenes, xanthoangelol and 4-hydroxyderricin, for enhanced osteosarcoma immunotherapy.^246–250^ These compounds all effectively inhibited the activation and differentiation of type 2 macrophages to suppress osteosarcoma cell proliferation and metastasis.

### Osteosarcoma-associated vaccines

**Fig. 6:**
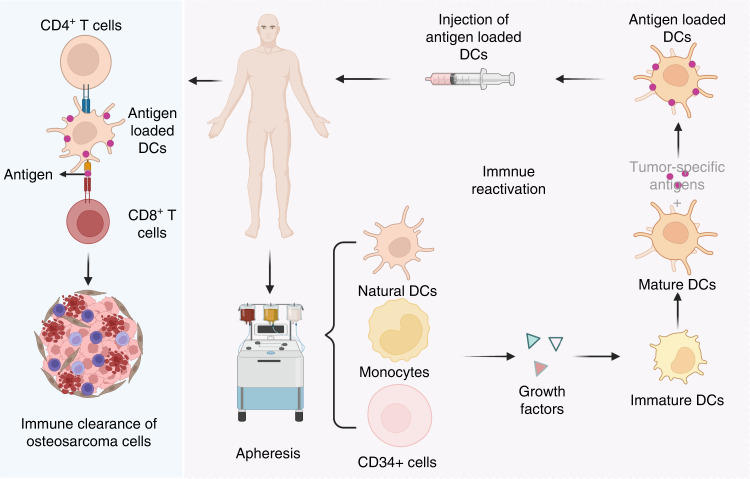
The main process used to generate ex vivo DC vaccines. Progenitor cells are extracted from patients via dialysis, induced to transition into mature DCs, and then loaded with tumor antigens for administration to patients. Tumor antigen-specific T lymphocytes are activated by injected antigen-loaded DCs to exert an antitumor effect

Osteosarcoma-associated vaccines are considered a novel immunotherapy method that exerts antitumor effects by activating a patient’s own endogenous immunity (Fig. 6). Studies on tumor antigens utilizing tumor-relevant substances composed of tumor proteins or peptides, autologous DCs, gangliosides, and autologous or allogeneic cancer cells to activate systemic immunity are ongoing.^251–253^ Adjuvants, cytokines, and other immunomodulators have also been applied in vaccine preparation to improve antitumor immunity.^82^ For instance, autologous tumor lysates were first used as tumor-associated vaccines and significantly prolonged the overall survival time of patients with osteosarcoma.^254^ Immune responses to peptides derived from the TAA papillomavirus binding factor (PBF) were found in 9 of 11 patients with refractory osteosarcoma.^83^ Moreover, T lymphocyte responses were detected in 20 of 28 patients with osteosarcoma and were related to long DFS (more than 5 years) in 2 patients with an anti-idiotypic antibody who received vaccination.^84^ The use of tumor vaccines is gaining momentum. Tumor vaccines are mainly divided into autologous cancer cell- and immune cell-based vaccines and noncell-based vaccines. Of these types, immune cell-based vaccines take full advantage of the activation of effector T lymphocytes by innate immunocytes, such as macrophages, DCs, and γδT lymphocytes. At the same time, however, the feasibility of modulating migration and activation is a major concern, as these processes are regulated by immunoinhibitory substances in the tumor microenvironment and the quantity and quality of compromised immune effector cells in patients.^255^ On the other hand, autologous cancer cell- and noncell-based vaccines have shown potential clinical value, as they can circumvent these barriers. It should be noted that the mechanisms of action of autologous cancer cell-based vaccines are independent of HLA-I, and the function of the patients’ immune system is to specifically select the most immunogenic antigen.^256^ Noncell-based vaccines generally have better antitumor effects and biocompatibility since they do not induce off-target effects.^257^

For example, melanoma-associated antigen 1 (MAGE-A1) was isolated and the first HLA-restricted anti-MAGE-A1 T lymphocytes and epitopes were identified using autologous CTL identification.^258^ Canine osteosarcomas are the only other spontaneous osteosarcomas in large animals, and their immune infiltrates resemble those of human osteosarcomas.^90^ It should be noted that mifamurtide (MTP-PE), the most effective systemic agent in canine osteosarcoma, has been approved in Europe and Japan for clinical use.^90^ Another example, DXS31-164, a Listeria monocytogenes vaccine (recombinant Listeria monocytogenes expressing a chimeric human HER2/neu construct), effectively triggers a HER2/neu-specific immune response to suppress pulmonary metastasis and prolong overall survival in a canine osteosarcoma model.^259^ The study also suggested the excellent effects of DXS31-164 in the treatment of HER2/neu-positive osteosarcoma patients. A recent study showed that the human anti-idiotypic vaccine 105AD7 was effective in most young osteosarcoma patients in clinical trials, without notable side effects.^260^ Another study also demonstrated that this vaccine could effectively elicit T-lymphocyte-mediated immunity in osteosarcoma patients and target a natural antigen (CD55) with amino acid and structural homology with 105AD7.^261^ Furthermore, various trials have also suggested that protein-based tumor vaccines, including those composed of tumor-rejection antigens and papillomavirus binding factors, can be used for specific immunotherapy in osteosarcoma and other malignant cancers due to shared overexpressed TAAs.^262–267^

DCs, considered professional APCs, make up approximately 0.3% of the total cell population in the blood and can promote the proliferation and differentiation of CTLs.^268,269^ Moreover, the quantity of DCs is strongly associated with the survival time of patients.^270,271^ Therefore, DC-based vaccines are the most common vaccination approach for pediatric sarcomas, and patients who received a DC vaccine had a prolonged survival time compared with control patients.^272,273^ In some encouraging cases, patients with recurrent or metastatic Ewing’s sarcoma, even in the presence of chemotherapy-induced immune cell loss, have shown significant improvement and extended survival time after receiving immunotherapy such as the DC vaccines, influenza vaccines, and autologous T lymphocytes.^274^ Cancer-associated vaccines have effectively enhanced T lymphocyte-mediated immunity even after treatment with chemotherapy or radiotherapy, but they have not yet achieved satisfactory effects in the treatment of most solid tumors, including osteosarcoma.^275^ Only one in ten patients showed a complete immune response while most patients showed progression in a phase I trial of a combined decitabine/DC vaccine for neuroblastoma and sarcoma.^276^ Considering the complex tumor microenvironment, this low level of response might be due to the interactions between various immune cells, including infiltrating type 2 macrophages, and the imbalance of Treg and Th17 cells.^277^ Consequently, further research in this area is still needed, with a focus on addressing the limitations of tumor vaccines in the immune microenvironment of osteosarcoma.

### Osteosarcoma-associated cytokines

Recently, a growing number of reports have demonstrated that cytokines can activate cells in response to immunotherapy to improve the immune response, and some proinflammatory cytokines (such as IL-8 and TNF-α) and immunosuppressants are closely associated with osteosarcoma progression.^278–280^ Thus, unsurprisingly, cytokine-based treatments have been commonly used in tumor immunotherapy for over three decades due to their wide range of effects, such as activating T lymphocytes and regulating antigen presentation.^281–283^ Moreover, cytokines secreted by TAMs and mast cells can manage immune cells and activate the inflammatory response to foreign antigens during invasion.^284^ Among these cytokines, ILs play a significant role in the expression of cellular adhesion molecules (CAMs), which are involved in the binding of NK cells to targets.^77,285^

Activating immune-modulating cytokines, such as IL-12, can facilitate T or B lymphocyte maturation, proliferation, differentiation, and antibody production as well as activate CILs, NK cells, and other immune effector cells and have shown significant curative effects in melanoma and kidney cancer.^286–288^ For example, IL-12 plays a significant role in decreasing side effects, such as gastrointestinal bleeding caused by chemotherapeutic or radiotherapeutic agents, and increasing patient tolerance.^289^ Moreover, IL-15-activated NK cells can effectively lyse tumor cells in high-grade osteosarcoma patients, particularly when the NK cells are activated by other cytokines.^290^ In addition, a prospective study of patients with primary metastatic osteosarcoma showed a survival of more than 40% for patients at 3 years after treatment with chemotherapeutic agents and IL-2. This result is encouraging and higher than that reported elsewhere.^291^ However, there remain limitations to the clinical application of cytokines due to the adverse effects resulting from the overactivation of immune responses caused by administering high concentrations of cytokines.^292,293^ In Table 3, we have summarized recent preclinical studies on cytokines, vaccines, and other immunotherapies for osteosarcoma.

**Table 3:** Preclinical immunotherapies in osteosarcoma

Approach	Models	Key elements	Reference
Cytokines	Osteosarcoma cell lines, mice	IL-34, IL18, IL12	^480–482^
CAR-T cells	Osteosarcoma cell lines, mice	CD166.BBζ, CAR-T cells, Sleeping Beauty-based components, CD8^+^ T cells, HER2-specific T cells	^48,353,483^
Vaccines	Osteosarcoma cell lines, DCs, mice	CD103^+^ dendritic cells, novel oncolytic vaccinia viruses, virus B1 kinase, tumor vaccines containing B7-1-transfected cells	^48,361^
Others	Osteosarcoma cell lines, mice, macrophages	Macrophage polarization	^476,484^

### Immune checkpoint blockade-based immunotherapy for osteosarcoma

Recently, some monoclonal antibodies, such as those targeting CTLA-4, B7-H3, PD-1, and the PD-1 ligand PD-L1, have been designed to block immune checkpoints and have attracted much interest because of their satisfactory antitumor efficicacy.^294–298^ However, clinical trial results to date suggest that most checkpoint blockers are less effective in treating solid tumors, including osteosarcoma.^275^ The reason for this is not completely understood, and it is possible that T lymphocytes are not the main effector cells inhibiting osteosarcoma in humans. A study detecting PD-1, PD-L1, and PD-L2 expression in 234 clinical samples from patients with musculoskeletal tumors reported that PD-1 and PD-L1 were negatively associated with prognosis and overall survival in osteosarcoma patients.^19^ This study also suggested that nivolumab, a PD-1 inhibitor, increased the proportions of CD4^+^ and CD8^+^ T lymphocytes and improved the cytotoxicity of CD8^+^ T lymphocytes to effectively suppress pulmonary metastasis of osteosarcoma in in vivo assays. Moreover, M2-like TAMs were reprogrammed and pulmonary metastasis of osteosarcoma was inhibited by decreasing the expression of PD-1.^299^ The current understanding of the mechanism of immune checkpoint blockers and the interaction between immune and osteosarcoma cells are summarized in Fig. 3.

In addition, the proportion of Tregs is significant increased in some tumor patients, and Tregs are considered important contributors to escape of immunological surveillance and poor immunotherapy outcomes.^300–302^ Hence, decreasing the quantity of Tregs is one of the main goals for Treg-based antitumor immunotherapy.^303^ For instance, anti-CD25 monoclonal antibodies have been used to reduce the number of CD4^+^ and CD25^+^ Tregs to promote specific CD8^+^ T lymphocyte-mediated cytotoxicity in breast cancer.^304,305^ In addition, blocking Tregs to modulate their immunosuppressive functions is also considered another effective strategy. Generally, ligands associated with immunosuppression are highly expressed on the Treg surface, including PD-L1, CTLA-4, and glucocorticoid-induced TNF receptor (GITR), which can be effectively blocked by specific inhibitors.^174,306–308^ Furthermore, enhancing the function of effector T lymphocytes to reverse the suppressive effect of Tregs has also been used in tumor immunotherapy.^309^ These ligands, which act as a brake for T lymphocytes, mainly induce TILs involved in antitumor immunity that fail to eliminate osteosarcoma cells, while related inhibitors can enhance T lymphocyte-induced antitumor immunity by MHC presentation to overcome obstacles and reverse this process.^310,311^

Notably, strategies aimed at the novel targets PD-1 and CTLA-4 represent a new era of antitumor immunotherapy and improve the potential for osteosarcoma therapy. PD-1 is a type 1 transmembrane protein that is usually found on the surface of activating effector T lymphocytes, B lymphocytes, and NK cells. It can interact with PD-L1 existing on the surface of tumor-infiltrating lymphocytes (TILs) and tumor cells. Strategies that suppress this interaction have shown significant therapeutic effects in cancer patients, including osteosarcoma patients.^312,313^ The expression levels of PD-L1 vary widely among osteosarcoma cell lines but are higher than those in parental cell lines.^314^ Moreover, the expression of PD-L1 shows a positive association with chemotherapy resistance and the quantity of TILs as well as with osteosarcoma cell proliferation.^315,316^ Consequently, high expression levels of PD-L1 are associated with worse survival time than low PD-L1 expression in osteosarcoma patients. Blocking the interaction of PD-1/PD-L1 may be a potential strategy to enhance the T lymphocyte-mediated immune response to improve osteosarcoma immunotherapy.

B7-H3 (also named CD276) is a member of the B7 family of molecules that can interact with CTLA-4 or PD-1.^317,318^ It is also generated in healthy tissues but is overexpressed in various tumor types, including osteosarcoma, and high CD276 expression is closely related to increased quantities of Tregs across tumor tissues, suggesting that CD276 has a positive effect on Treg-mediated suppression of T lymphocyte function.^319–322^ In a lymphoma mouse model, Lee *et al*. found that blocking the immune checkpoint CD276 effectively inhibited tumor progression, and combination of this strategy with an anti-PD-1 antibody led to further improved therapeutic efficacy in advanced tumors.^318^ Additionally, several preclinical reports have also shown the potential of using CAR-T lymphocytes or microRNAs (such as miR-124) to target B7-H3 to improve osteosarcoma immunotherapy.^323–327^ Recently, an orthotopic, spontaneously metastasizing osteosarcoma model was designed to predict the biocompatibility and efficacy of a new generation CAR-T-cell treatment and other therapeutic approaches for osteosarcoma metastasis.^320^ This model suggested that B7-H3 was highly and homogenously expressed in pediatric solid tumors. The CAR-T-cell strategy showed robust activity against various xenograft tumor models, and its efficacy was highly dependent on the density of TAAs. Notably, a phase I clinical trial using enoblituzumab (MGA271), considered a first-line monoclonal antibody, is in progress (NCT02982941) in adolescents with B7-H3-expressing osteosarcoma.^328,329^

CTLA-4 (also named CD152), a type 1 transmembrane glycoprotein receptor, is also expressed on Tregs and memory T lymphocytes and can bind to and compete with CD80 and CD86 on DCs due to their shared B7 ligands.^330–333^ Mechanistically, CTLA-4 can induce IDO to suppress T lymphocyte proliferation and cytokine secretion, leading to immunosuppression.^334,335^ It should be noted that ipilimumab, a human IgG4 monoclonal antibody, has been approved by the U.S. Food and Drug Administration (FDA) as a new generation immune checkpoint inhibitor against melanoma.^336^ Emerging evidence suggests that the risk of osteosarcoma is positively correlated with the expression of CTLA-4.^337–341^ Thus, CTLA-4 blocking agents and antagonists can restore antitumor immunity by activating B7 and CD28 signaling and depleting Tregs.^342^ In a phase I clinical trial in pediatric osteosarcoma, 25% of osteosarcoma patients who received ipilimumab developed stable disease with acceptable immune-associated side effects.^343^ However, the major issue with ipilimumab in pediatric patients is gastrointestinal side effects; therefore, safer and more effective immune checkpoint inhibitors and immunotherapies are urgently needed in the future. Table 4 summarizes preclinical studies of osteosarcoma immunotherapy.

**Table 4:** Current immune checkpoint blockade therapies

Therapeutic agent	Target site	Models	Clinical trial stage	Reference
Pembrolizumab	PD-1 inhibitor	Recurrent or progressed osteosarcoma	Single-arm, open-label, phase 2 trial	^485^
Nivolumab	PD-1 inhibitor	Recurrent or refractory osteosarcoma	Multicenter, open-label, single arm, phase 1–2 trial	^486^
Atezolizumab	PD-L1 inhibitor	Progressive osteosarcoma	Multicenter phase 1–2 study	^487^
Apatinib+ camrelizumab	PD-1 inhibitor+ tyrosine kinase inhibitor	Patients (≥11 y) with metastatic or locally advanced osteosarcoma	Single-arm, open-label, phase 2 trial	^488^
Anti-PD-1/CTLA-4 antibodies+ Bempegaldesleukin	PD-1/CTLA-4 inhibitor+ CD122- preferential IL-2 pathway agonist	Disseminated K7M2-WT metastatic osteosarcoma mouse model, K7M3 primary tibial osteosarcoma mouse model, and DLM8 subcutaneous osteosarcoma mouse model	Bempegaldesleukin (BEMPEG; NKTR-214) efficacy as a single agent and in combination with checkpoint inhibitor therapy in mouse models of osteosarcoma	^489^
Nivolumab+ ipilimumab	PD-1 inhibitor+ CTLA-4 inhibitor	Locally advanced, unresectable, or metastatic osteosarcoma	Two open-label, noncomparative, randomized, phase 2 trials	^490^

### Adoptive cell therapy for osteosarcoma

Active and adoptive immunotherapies are considered two immunotherapy formats for tumor treatment.^344,345^ The former, including DCs, pulsed vaccines, and cytokines, can effectively activate immune responses against cancer cells.^346,347^ Adoptive immunotherapy, considered passive immunotherapy, refers to the injection of in vitro-expanded cancer-specific cytotoxic immune cells, particularly T lymphocytes.^63,348,349^ Adoptive cell therapy (ACT) involves the collection of self immune cells, including NK cells and TILs, which are expanded in ex vivo culture and induced to express chimeric antigen receptors (CARs) or T lymphocyte receptors (TCRs) for ACT.^350^ The three main ACT types are CAR-modified T lymphocytes, TCR-modified T lymphocytes, and TILs. These approaches circumvent the shortcomings of both immune checkpoint blockade-targeted T lymphocyte activation and vaccine approaches due to their high affinity for specific TAAs and lack of a requirement for peptide recognition in the context of HLA. They have fewer side effects than chemotherapy.^351^

HER-2 receptors are expressed in 40% to 60% of primary osteosarcoma patients but at a low level. The outcome and biocompatibility of HER2-based CAR-T therapy have been investigated in trials in HER2-positive sarcoma, and dose-limiting toxicity has not been observed.^352–354^ Although it is expressed at a lower level, HER2 can also be efficiently recognized by CAR-T lymphocytes, suggesting that CAR-T cells have great potential to target cancer cells. By using an osteosarcoma mouse model, researchers found that metastatic cancer cells that were insensitive to chemotherapeutic agents could be efficiently eliminated by the IL-11 receptor α-chain (IL-11Rα) and CAR-T cells modified to target HER2.^355^ In a recent report, a cell membrane-modified and site-specific IL-12 (attIL12) was used to engineer peripheral blood mononuclear cells (PBMCs) rather than T lymphocytes to omit the expansion phase of the desired CAR-T cells.^356^ These IL12-targeted attIL12-PBMCs induced an observable antitumor effect in both heterogeneous osteosarcoma patient-derived xenograft tumors and metastatic osteosarcoma models with no significant side effects. Satisfactory outcomes of adoptive T lymphocyte transfer and ACT in osteosarcoma and other cancers have been reported in previous studies.^357–360^ As an example, CD166-specific T cells were obtained by virus-based transfer of the corresponding DNA plasmids. These cells were selectively expanded using IL-2 and IL-15, and the ability of CD166.BBζ CAR-T cells to kill CD166^+^ osteosarcoma cells was evaluated in vitro and in vivo. The CD166.BBζ CAR-T cells killed osteosarcoma cell lines in vitro, and the cytotoxicity degree was correlated with the levels of CD166 expression on the tumor cells. Intravenous injection of CD166.BBζ CAR-T cells into mice caused tumor regression with no obvious toxicity.^361^ These successful in vivo studies support further exploration, especially with regard to improving the outcomes of CD166-related therapies. However, safety-related modifications to avoid potential adverse effects of CAR-T treatment must be considered. The CD166-targeted T lymphocyte treatment discussed above represents a clinically appealing strategy for osteosarcoma patients with positive CD166 expression, offering a starting point for additional investigations of clinical osteosarcoma immunotherapy.

Unlike CAR-T-cell therapy, TCR T lymphocytes are generated from high-affinity and highly acidic TAA-specific T-lymphocyte clones, increasing the specificity and sensitivity of TCR T cells for targeting cell surface HLA.^362^ TCR T cells show excellent targeting efficacy compared to CARs or antibodies, penetrating tumors and binding to HLA-presented tumor intracellular and surface antigenic peptides.^363^ TILs are generally collected from resected tumors, followed by in vitro proliferation, and are then administered to subjects in considerable quantities.^364^ However, the strategies for isolation of TILs from osteosarcoma tissues and induction of their proliferation are not yet completely optimized, as the number of TILs acquired is frequently not adequate for immunotherapy.^365^ Another challenge in the treatment of osteosarcoma is the few immunoregulatory factors and excess inhibitory substances of osteosarcoma cells, which might suppress the activation and expansion of TILs.^364^ A new generation of TILs has shown good long-term persistence, which is consistent with the memory phenotypes of most T lymphocytes. For example, a novel therapy based on lifileucel (LN-144), an autologous, tumor-infiltrating lymphocyte product, was reported to induce a durable response and have excellent disease control in melanoma patients who had previously failed ICB therapy.^366^ In addition, ICB combined with TIL-based therapy may also be an effective therapeutic strategy for osteosarcoma patients who have progressed on monotherapy. Antagonistic anti-CTLA-4 antibodies have been verified to improve the HLA binding ability of TILs in melanoma as well as facilitate CD8^+^ TIL proliferation in Lewis lung carcinoma.^256^ A recent study also revealed that TIL-based strategies in combination with anti-PD1 antibody therapy showed excellent therapeutic efficacy in patients with pulmonary metastasis of osteosarcoma.^367^

### Immunotherapy combinations for osteosarcoma

Recently, emerging evidence has shown that the expression level of PD-1 on CD8-positive TILs is greatly reduced after treatment with an anti-PD-L1 antibody, while the expression of CTLA-4 is increased in a metastatic osteosarcoma mouse model, indicating that PD-L1 and CTLA-4 may have complementary roles in inhibiting CD8^+^ CTL-induced antitumor immunity.^368^ These results provide enthusiasm for immunotherapy studies and the use of combination approaches. For example, the combination of α-PD-L1 and α-CTLA-4 antibody blockade leads to enhanced suppression of osteosarcoma metastasis and maintains osteosarcoma immunological surveillance in mouse models.^368^ In a clinical trial, approximately 30% of cancer patients were alive with a median of 13.6 months of follow-up in the nivolumab monotherapy arm, and the survival of half of the patients was significantly extended with the combination of ipilimumab and nivolumab therapy.^369^ Administration of anti-CTLA-4 antibody therapy combined with other immune stimulants, such as cryotreated tumor lysate-pulsed DCs, achieved increased antitumor immune responses in a murine osteosarcoma model.^56^ ICB antagonists are also able to improve the cytotoxicity of CAR-T lymphocytes and bispecific T-lymphocyte engager (BiTE) antibodies, resulting in reversal of the immunosuppression induced by released immunosuppressive factors and the lack of specific TAA presentation; these effects reactivate inhibitory and exhausted T lymphocytes.^370–372^ Encouraging outcomes from combination therapy with checkpoint antagonists and genetically engineered T lymphocytes or cancer vaccines have also been shown in phase I trials.^373–375^ As an example, the combination of CTLA-4 blockade and IL-21-activated polyclonal antigen-specific CTLs effectively inhibited melanoma metastasis in refractory patients, and this approach was shown to be biocompatible and achieved durable immunity in another patient, giving new hope for osteosarcoma immunotherapy.^376,377^

Combinational treatments with PD-L1/PD-1 blockade, CTLA-4 inhibitors, and small molecule IDO inhibitors have also resulted in strongly enhanced tumor suppression in multiple tumor models (including osteosarcoma models) because they expand infiltrating T lymphocytes and decrease Treg and MDSC numbers.^378–380^ For example, the combination of pembrolizumab and epacadostat has been generally well tolerated and presented satisfactory anticancer effects in various advanced cancers (such as endometrial cancer, kidney cancer, and melanoma) in multicenter clinical trials.^381^ However, pembrolizumab in combination with epacadostat did not extend progression-free survival or overall survival compared to placebo in combination with pembrolizumab in patients with unresectable or metastatic melanoma, indicating that IDO1 suppression may not be an effective strategy to improve anti-PD-1 therapy.^382^ However, adoptive ex vivo-expanded γδT lymphocyte transfer, especially combined with immunostimulants such as amino bisphosphonates, could be a promising approach for osteosarcoma immunotherapy.

Monoclonal antibodies, such as anti-GD2 antibodies, have also been found to improve antitumor immunity in combination with colony-stimulating factor 2 (CSF2) or IL-2 in solid tumors^383,384^ For example, some studies have reported that anti-ganglioside GD2 monoclonal antibodies can synergize with chemotherapeutic agents to cause endoplasmic reticulum (ER) stress-related cell death.^385,386^ Trials exploring checkpoint blockers in combination with adjuvant and neoadjuvant chemotherapy or site-specific treatment are also ongoing, such as studies assessing camrelizumab in combination with apatinib (NCT03359018) or pembrolizumab in combination with axitinib.^312,387,388^ These combination treatments may play a beneficial role during chemotherapy by decreasing tumor burden, exposing neoantigens by inducing tumor necrosis, and directly attacking tumor stromal cells.^389^ For instance, the preoperative chemotherapeutic agent ifosfamide in combination with the immunotherapeutic agent IL-18 can efficiently suppress the progression of pulmonary metastases in a murine osteosarcoma model.^390^ Another study also reported that the combination of α-PD-L1 antibody and L-arginine can effectively enhance anti-osteosarcoma immunity and greatly extend survival time.^391^ Some immune cells, such as DCs, have been shown to be efficacious in osteosarcoma therapy in combination with low-concentration chemotherapeutic agents, such as doxorubicin (DOX), which can effectively induce ICD to further activate immunity against osteosarcoma.^392,393^ CAR-T lymphocytes combined with antibodies and other T lymphocyte-based treatments, such as adoptive T-lymphocyte transfer, are also becoming increasingly common.^394^ Overall, combination immunotherapy can boost the duration of the immune response because the double-agent treatment activates antitumor immune memory effects in osteosarcoma patients who do not respond to monotherapy. Advances in both the understanding of immunotherapy and relevant technologies and further exploration of combination treatments in clinical trials will further boost therapeutic efficiency for patients with osteosarcoma.

## Nanotechnology-based immunotherapy for osteosarcoma

**Fig. 7:**
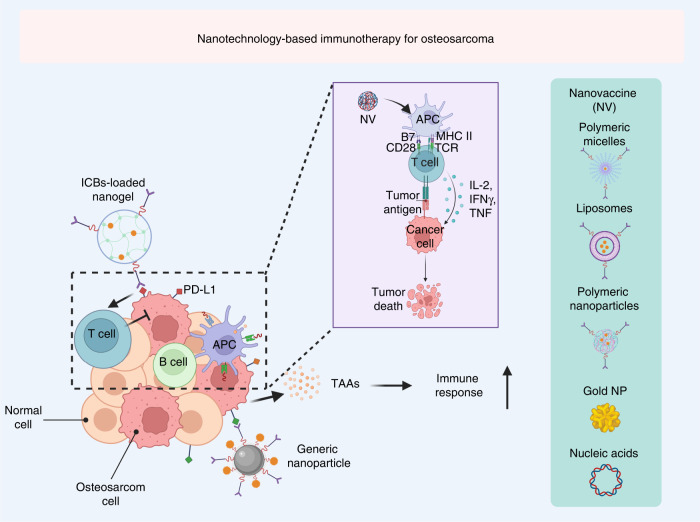
Schematic illustration of nanotechnology-based immunotherapy for osteosarcoma

Some synthetic and natural polymer materials have shown immunomodulatory activity in the absence of external factors. For example, these materials can repolarize immunosuppressive type 1 TAMs into immune-supportive type 2 TAMs and reverse the immunosuppressive microenvironment of osteosarcoma. These materials generally have special physical properties, such as properties related to energy radiation and absorption, which can be harnessed to eliminate cancer cells, as in imaging-guided nanomedicine. Moreover, these nanoparticles have good optical and magnetic profiles and can be applied for photodynamic therapy (PDT), photothermal therapy (PTT), radiotherapy, and magnetothermal therapy in response to exogenous activation to induce immunogenic cell death (ICD) and augment antitumor immunity (Fig. 7). Furthermore, versatile nanoplatforms have been developed that can be rationally applied in living animals to deliver agents to specific sites or perform precise functions, which may be applied in the treatment of osteosarcomas that are resistant to immunotherapy, chemotherapy, and radiotherapy (Table 5). Consequently, nanoparticles can greatly facilitate the delivery of various anti-osteosarcoma agents, and relevant studies will provide new insights and strategies to destroy osteosarcoma cells with immunotherapy.

**Table 5:** Examples of different nanoparticles for osteosarcoma therapy

Nanoplatform	Name	Therapeutic agent	Therapeutic application	Reference
Liposomes	Chol-SS-mPEG/HA-L	DOX	Chemotherapy	^491^
Hydrogels	SP@MX-TOB/GelMA	Tobramycin (TOB)	Chemotherapy, photothermal therapy	^492^
Chitosan	ZSM-5/CS/DOX nanodisks	DOX	Chemotherapy	^493^
Nano-oxides	PEG-GOFA/ICG	DOX and TH287	Chemotherapy, photodynamic therapy	^494^
Nanocomposite oxides	β-TCP-Fe-GO	Fe3O4 magnetic particles	Hyperthermal therapy	^495^
Nanometals and nanoalloys	Au@AgNRs@BSA	Au and Ag nanoparticles	Photothermal therapy	^496^
Carbon nanotubes	SWCNTs	Single-walled carbon nanotube	Chemotherapy	^497^
Micelles	NP-PTX-DOX	PTX and DOX	Chemotherapy	^498^
Dendritic macromolecules	AG/G5-Dox NGs	DOX	Chemotherapy	^499^
Nanocapsules	IFS-LNC	Ifosfamide	Chemotherapy	^500^
Exosomes	EXO-DOX	DOX	Chemotherapy	^501^

### Targeting cancer cells for immunogenic cell death

Recently, a growing number of studies have reported that dying cancer cells can release immunoregulatory damage-associated molecular patterns (DAMPs) to effectively counteract specific or nonspecific antitumor immunity in immunotherapy.^395,396^ This promotion of cancer cell death by activating immunity is also referred to as immunogenic cell death (ICD).^397,398^ It is closely related to DAMPs, such as adenylate triphosphate (ATP), high mobility group protein B1 (HMGB1), surface calreticulin (CAT), and heat shock protein 70 (HSP70), which can mediate DC or TAM changes to improve antigen presentation and immune infiltration for effective immunotherapy.^399,400^ Moreover, the dying tumor cells can also release TAAs to recruit immune cells, and these TAAs serve as promising therapeutic targets for immunotherapy.^401^ Notably, versatile nanotechnology-based nanodelivery platforms are considered effective tools for inducing ICD because they can deliver useful concentrations of cytotoxic agents to cancer cells, resulting in improvements in the rate of ICD induced by different therapies, such as radiotherapy, chemotherapy, and phototherapy.^31,402^

#### Chemotherapy/radiotherapy

A number of reports have shown that dying osteosarcoma cells are immunogenic and can enhance immunity against osteosarcoma after treatment with certain chemotherapies or radiotherapy.^393,403^ Anthracyclines, including doxorubicin (DOX), mitoxantrone (MTO), oxaliplatin, bortezomib, and cyclophosphamide, are the most classic ICD inducers.^404^ For instance, a mitochondria-targeted nanomicelle (named OPDEA-PDCA) capable of initiating mitochondrial oxidative stress was designed to induce pyroptosis of osteosarcoma cells. In this nanoplatform, poly[2-(N-oxide-N,N-diethylamino)ethyl methacrylate] (OPDEA) is employed to target mitochondria, and modified dichloroacetate (DCA) is employed to suppress pyruvate dehydrogenase kinase 1 (PDHK1) to induce mitochondrial oxidative stress, which can lead to pyroptosis and further mediate ICD in osteosarcoma cell lines.^405^ However, OPDEA-PDCA can also promote the release of soluble PD-L1. Thus, OPDEA-PDCA in combination with an anti-PD-L1 monoclonal antibody substantially inhibits osteosarcoma cell proliferation and enhances T lymphocyte activation. In addition, a pH-responsive autophagy-regulated nanoplatform (named CBZP), which encapsulates both the natural product curcumin (CUR) to enhance the antitumor immunity of PD-1/PD-L1 blockade by mediating autophagic cell death-mediated ICD and the immune checkpoint inhibitor BMS1166 to simultaneously suppress the PD-1/PD-L1 interaction to improve tumor immunogenicity and improve T lymphocyte immunity, has also been developed for osteosarcoma immunotherapy.^406^ After being taken up by osteosarcoma cells, the pH-responsive nanoplatform triggers autophagy and increases the intracellular acidic environment, which in turn further promotes the release of CUR to augment autophagic activity. Importantly, administration of CBZP to orthotopic osteosarcoma-bearing mice presented excellent antitumor efficacy and achieved a long-term immune response to inhibit tumor recurrence, which was also accompanied by improved DC maturation and tumor infiltration of CD8^+^ T lymphocytes. In summary, this nanoplatform has shown beneficial anti-osteosarcoma effects by integrating an agent to induce ICD activation and immune checkpoint inhibitors, thus shedding light on the use of autophagy modulation as a promising modality for osteosarcoma treatment.

#### Phototherapy

Near-infrared (NIR) laser-activated phototherapy has been widely used in basic research of osteosarcoma.^34,407–409^ PTT mainly relies on a high local temperature to inhibit tumor cell proliferation, which can effectively eliminate cancer cells and enhance antitumor immune responses.^410–412^ Moreover, PTT driven by nanoparticles has also been shown to induce effective treatment outcomes and is able to facilitate local heating of tumor tissues without impairing the surrounding normal tissue.^413^ Additionally, PDT, as another noninvasive therapeutic approach, can also be activated by a laser of a specific wavelength, resulting in cytotoxic ROS accumulation in the presence of endogenous O_2_ to eventually induce the death of tumor cells.^414–416^ Notably, studies have reported that cancer cells can also release TAAs after laser exposure, promoting the secretion of cytokines and the maturation of DCs, which helps them activate T lymphocytes to induce robust antitumor immunity.^417,418^ These results suggest that dying cancer cells releasing TAAs after phototherapy can induce adjuvant effects by stimulating an immune response, and thus, this strategy can be considered an “automatic vaccine”.^419,420^ For instance, a tumor cell membrane-modified Au nanoplatform (named C-RAuNC) was encapsulated with the ferroptosis agonist RSL3 and administered to prevent osteosarcoma drug resistance.^421^ In this nanoplatform, RSL3 can inhibit glutathione peroxidase (GPX4) to mediate ferroptosis. Moreover, Au nanoparticles encapsulated in tumor cell membranes can achieve site-specific delivery, controlled drug release, effective phototherapy, and amplified ICD to enhance the immune response and thus overcome osteosarcoma drug resistance. In another example, encouraging results of therapy for osteosarcoma drug resistance were achieved by C-R-AuNCs, which are believed to be a promising modality for the future clinical treatment of drug-resistant osteosarcoma. In PDT research on osteosarcoma, increased expression of HSP70 has been shown in the MG-63 cell line, which is in line with enhancement of immunity and indicates the promise of ICD induced by PDT in osteosarcoma therapy.^422^

#### Chemodynamic therapy

In addition to the above approaches, chemodynamic therapy (CDT) can also improve immunogenicity for osteosarcoma immunotherapy.^423,424^ CDT, a noninvasive treatment with a high tissue penetration depth compared to phototherapy strategies, can generate ROS via Fenton or Fenton-like reactions or sonosensitizers to induce tumor ablation, enhance immunogenicity, and achieve better antitumor immunity.^424–426^ For instance, alendronate (ALD)/K7M2 cell membrane-encapsulated hollow manganese dioxide (HMnO_2_) nanoplatforms were used as nanocarriers to encapsulate ginsenoside Rh2 (Rh2) for magnetic resonance imaging (MRI)-guided chemodynamic-immunotherapy combination treatment in osteosarcoma.^427^ ALD and K7M2 cell membranes were successively modified on the surface of the nanoparticles and encapsulated with Rh2. This tumor microenvironment-responsive nanoplatform showed good osteosarcoma-targeting and homing abilities, excellent GSH-responsive drug release and an excellent MRI profile, and useful chemodynamic-immunotherapy combination treatment effects. These designed nanoplatforms can efficiently induce ICD, activate CD4^+^/CD8^+^ T lymphocytes in vivo, and promote the expression of BAX, BCL-2 and Caspase-3 at the cellular level to improve antitumor immunity. Further results have suggested that these nanoparticles improve the release of TNF-α, IFN-γ, and IL-6 into the serum and suppress the production of FOXP3^+^ T lymphocytes in tumors. Furthermore, these nanoplatforms greatly inhibit tumor cell proliferation in situ in tumor-bearing mice. Consequently, the combination of such efficient and biocompatible nanoplatforms with immunotherapy and chemodynamic therapy will likely produce excellent strategies for osteosarcoma treatment.

### Targeting DCs for enhanced osteosarcoma immunotherapy

Capsaicin, a chemotherapeutic agent, can effectively induce ICD to improve the DC-mediated phagocytosis of MG-63 cells and the presentation of relevant antigens to enhance the immune response.^428^ Additionally, the liposomal-muramyl tripeptide phosphatidylethanolamine, not only as monotherapy but also in combination with other therapeutic agents, can also activate DCs and stimulate T lymphocyte proliferation to extend overall survival without inducing metastasis.^429^ Moreover, efficient site-specific delivery of immunomodulators or immunostimulatory factors to innate immune cells (i.e., DCs) or tumor-draining lymph nodes is useful for enhancing the immune response for nanovaccines.^430^ Generally, almost all types of delivery platforms, such as nucleic acids, polymeric micelles, polymer nanoparticles, liposomes, and inorganic nanoparticles, can be rationally used for therapeutic nanovaccines.^431^ It should be noted that antigens can be in the form of recombinant proteins, synthetic long or short peptides, DNA, or RNA because nanocarriers can prevent cargo degradation.^432^ In addition, the most commonly studied nanoadjuvants in tumor vaccines, including oligodeoxynucleotides (ODNs), monophosphoryl lipid A (MPLA), 5′-C-phosphate-G-3′ (CpG), LPS, TLR agonists, polyinosinic: polycytidylic acid (poly I:C), and agonists of stimulator of IFN genes (STING), can efficiently augment antitumor immunity and greatly improve the therapeutic effect of these cancer vaccines.^433–435^ Similarly, this flexibility makes nanoscale vaccines suitable for boosting antitumor immunity, as they may mediate a robust cellular and humoral immune response in vivo. Moreover, these nanovaccines can not only be efficiently transported from the site of administration to the tumor-draining lymph nodes, triggering robust antitumor immunity and immune memory, but also boost DC maturation and accelerate the efficacy of targeting DCs, leading to stronger T lymphocyte-mediated antitumor immunity.^436–438^ Tuohy *et al*. developed a robust hyperthermia-based nanotherapy platform in a murine osteosarcoma model. The researchers found that osteosarcoma-bearing mice with osteomyelitis showed a higher proportion of “nonclassical” monocytes (Ly6Clo) than all other groups.^439^ There were significant changes in monocyte expression of various chemokine receptors, such as CXCR2, CCR2, and CXCR4, among the experimental groups. Monocytes from osteosarcoma-bearing mice treated with hyperthermia therapy showed greater chemotaxis than monocytes from osteosarcoma-bearing mice with osteomyelitis. In addition, the tetrafunctional amphiphilic blocker copolymer 704 was used to deliver a fractalkine (FKN)-encoding plasmid to evaluate its antimetastatic effects.^440^ FKN was described as an excellent candidate to induce robust antitumor immunity in various tumors.

### Targeting the immunosuppressive microenvironment of osteosarcoma

Immunosuppressive factors in the tumor microenvironment act as the main substances that promote tumorigenesis and malignant progression.^441,442^ Strategies that modulate the immunosuppressive microenvironment of tumors can polarize immune cells to a phenotype suitable for antitumor immunity. Nanoparticles can be used for temporal and spatial modulation of Tregs, TAMs, TANs, and MDSCs as well as immunosuppressive soluble substances, presenting a potential strategy for transitioning cold tumors to hot tumors.^443–445^ Consequently, the site-specific, targeted and precise modulation of these tumor-relevant immunosuppressive cells should provide rationally designed nanoplatforms to effectively deliver immunotherapeutic agents to the tumor microenvironment in vivo.^446–448^

MDSCs mainly accumulate in peripheral lymphoid organs or tumor tissues and induce tumorigenesis, malignant progression, and metastasis by secreting immunosuppressive factors (such as IL-10, IDO, and arginase-1 (Arg-1)), activating Tregs and suppressing the activity of T lymphocytes and NK cells.^449–451^ Therefore, blocking, reprogramming, or depleting MDSCs has been considered a promising strategy for restoring the antitumor immune response.^452^ For instance, a recent study reported robust nanoparticles (HA/ZIF-8@Gem/D-1-MT NPs) that could efficiently encapsulate the chemotherapeutic agent gemcitabine and the IDO inhibitor 1-methyl-DL-tryptophan (1-MT) to deplete MDSCs and suppress IDO.^453^ Release of gemcitabine effectively induced encouraging chemotherapeutic effects on osteosarcoma cells with subsequent MDSC depletion, and IDO in osteosarcoma cells was suppressed by 1-MT, leading to synergistic antitumor immune effects. Efforts are underway to develop treatments that reduce tumor-related inflammation and the numbers of immunosuppressive cells. Another study suggested that a previously reported deep-tissue imaging strategy that uses indocyanine green-encapsulated calcium phosphosilicate nanoparticles (ICG-CPSNPs) could be used as an immunomodulatory agent to enhance antitumor immune responses. The theranostic application of ICG-CPSNPs as photosensitizers for PDT effectively inhibited tumor cell proliferation in a murine model of metastatic osteosarcoma by reducing inflammation-expanded immature myeloid cells. Consequently, this therapeutic approach was also named PhotoImmunoNanoTherapy.^454^

### Immune checkpoint blockade for osteosarcoma

Nanotechnology has provided a potential approach to overcome the side effects of ICB agents. For example, a recent study reported on a locally deliverable ICB nanogel that was injectable and NIR-sensitive and could be used for postsurgical osteosarcoma immunotherapy.^455^ This injectable nanogel with an adjustable solution-to-gel transition temperature was generated by copolymerization of thermosensitive and zwitterionic monomers. Subsequently, combined with multifunctional mesoporous nanomaterials, this nanocomposite could absorb NIR laser irradiation, leading to hyperthermia; this effect triggered a retro Diels-Alder (D-A) reaction to degrade the coating layer on nanoparticles, resulting in NIR-triggered drug release. Moreover, in an osteosarcoma postsurgical recurrence model, researchers found that this versatile delivery platform with favorable biocompatibility could effectively avoid early leakage of the drug and greatly enhance drug accumulation in tumors. In addition, the long-term controlled drug release of this nanoplatform greatly increased the quantity of active T lymphocytes, leading to a satisfactory antirecurrence effect. In summary, this study suggests that the locally injectable nanogel is a promising tool for postoperative treatment of patients with osteosarcoma. Moreover, another study also investigated the ability of PD-L1 downregulation with PDT combined with the autophagy inhibitor 3-methyladenine (3-MA) to prevent the long-distance metastasis of osteosarcoma.^456^ A significant tumor inhibition effect mediated by PDT was found in a partial resection model of osteosarcoma, revealing the potential clinical value of PDT during tumor surgery. At the same time, this study also found that the expression of PD-L1 was downregulated in tumor tissues in response to treatment with nanoparticles, which markedly enhanced antitumor immune responses to suppress osteosarcoma cell proliferation and metastasis. Notably, this immunological response triggered by the combination of the autophagy inhibitor and PDT inhibited osteosarcoma growth in in vitro and in vivo assays, suggesting the potential clinical utility of this strategy. Therefore, the combination of PDT with multimodal treatments may be promising for osteosarcoma immunotherapy.

### CAR-T cells for osteosarcoma immunotherapy

CAR-T cells have greatly influenced the current treatment landscape of hematological malignancies and shown significant clinical therapeutic benefits, and these positive results have generated enthusiasm for research in solid tumors, including osteosarcoma.^23,457–459^ However, the treatment of solid tumors faces a unique set of challenges in comparison with hematological tumors.^460,461^ For instance, the lack of selectively and uniformly generated TAAs and the immunosuppressive tumor microenvironment are considered the biggest obstacles to successful CAR-T-cell therapy.^462–464^ A combination of costimulatory factors and cytokines has been developed to improve CAR-T-cell activity and augment antitumor immunity. The expression of transgenic cytokines on CAR-T cells also enhances proliferative activity.^465^ At the same time, killing of T lymphocytes induced by redirected universal cytokines was achieved using a nuclear factor of activated T lymphocyte (NFAT)-responsive promoter that could promote the release of cytokines when TAAs were recognized by CARs, resulting in minimal side effects and increased cytokine concentrations in the tumor tissue.^466^ To promote migration to tumor sites, CAR-T cells have been engineered to coexpress CCL5 and CXCL9, which forms a feedback loop to amplify lymphocyte implantation via efficient CD8^+^ cell recruitment.^467^ Nanobiotechnology has been used to address these significant challenges to enhance the efficacy of CAR-T-cell therapy.^468^ For instance, a recent report showed that iron oxide nanoparticles can be used to label CAR-T cells for a clinically translatable approach, enabling noninvasive monitoring of the labeled CAR-T cells with magnetic particle imaging (MPI), photoacoustic imaging (PAT), and MRI for detection of their distribution in the body.^469^ The study showed enhanced nanoparticle internalization in CAR-T cells that did not affect their proliferation, viability, or function using a custom-made microfluidics device for T-cell labeling by mechanoporation. Multimodal imaging showed that T lymphocytes labeled with the Fe_2_O_3_ nanoparticles homed to osteosarcoma and off-target sites in mice, whereas no T lymphocytes were observed in groups treated with unlabeled cells. This manuscript details the successful labeling of CAR-T cells with ferumoxytol, opening up the possibility of detecting CAR-T cells in solid tumors. Moreover, a clinical trial (NCT04433221) that combined CAR-T cells with low-dose chemotherapeutic agents that are capable of regulating surface PD-L1 expression is in progress. Notably, CD28-based and CD28-CD3z-OX40 CAR-T-cell therapy have been used In clinical trials (NCT00902044 and NCT01953900) in sarcoma patients to enhance the costimulatory response. Importantly, multitarget CAR-T cells have also been developed to enhance TAA recognition and suppress the cancer recurrence induced by the overgrowth of certain TAA-negative cells or cells with low TAA expression.

## Conclusions and perspectives

Osteosarcoma is a primary malignant bone tumor, mainly develops in adolescence and has a poor prognosis. The immune microenvironment in osteosarcoma is complex, has high plasticity, and is closely associated with immune escape, uncontrolled proliferation, and metastasis of osteosarcoma cells. Therefore, remodeling the immunosuppressive microenvironment of osteosarcoma should be a strategy to remove small lesions and CTCs for efficacious osteosarcoma treatment. In this review, we systematically discussed the roles of various immune-associated cells in the immune microenvironment in osteosarcoma and the progress of relevant immunotherapy regimens and clinical applications. In particular, we highlighted the use of nanotechnology to modulate the immunosuppressive osteosarcoma microenvironment for enhanced osteosarcoma immunotherapy, thus providing guidance for the study, diagnosis, and treatment of osteosarcoma in the future.

Current immunotherapies for osteosarcoma, including tumor antigen vaccines, ICB strategies, and CAR-T therapy, have shown satisfactory therapeutic efficacy, but the promotion of metastasis by and immunosuppressive nature of the tumor microenvironment are still two major barriers to successful therapeutic outcomes. Overall, modulating the immune microenvironment of tumors to overcome immunosuppression and favor an antitumor immune response may be an important strategy for overcoming the current challenges of tumor therapy. A growing amount of evidence supports a comprehensive approach that can mediate antitumor immunity while overcoming the immunosuppressive tumor microenvironment, which should significantly boost clinical therapeutic outcomes.

Although such immunotherapy approaches have many advantages, there are also some disadvantages, including the extremely high cost, significant individual differences in immune responses, poor in vivo pharmacokinetic characteristics, and substantial adverse effects of systemic delivery, all of which need to be overcome. Thus, the research described above also has limitations. First, the immune microenvironment of osteosarcoma is complicated and dynamic and varies by disease type and duration and in different individuals. The current knowledge of the role of immune microenvironment components in osteosarcoma has been formed based on similar studies on other cancers, though the results may be both ambiguous and disease-specific. Moreover, most of the research is still in the preclinical stage, with insufficient evidence for translational medicine and nanotechnology-based drug delivery systems. Furthermore, antitumor immunity affects the whole body, and immune-associated treatments need to be both biocompatible and efficient. Such treatments may also result in new and serious side effects if not used properly; therefore, the side effects of these therapeutic agents must be seriously considered. Owing to strong genomic heterogeneity, targeted immunotherapy has not effectively improved overall survival for decades.^470^ Novel prognostic markers assessable at diagnosis are vital to identifying subsets of osteosarcoma. The focus of clinical trials has now shifted to serial phase II studies to evaluate the activity of novel agents in recurrent and refractory disease. In-depth analyses have revealed profound genomic instability and heterogeneity across patients, with nearly universal TP53 aberration. The complexity of the genome may support the role for immunotherapy.^471^ Characteristic gene expression classifiers can be applied to distinguish different categories of patients at initial diagnosis or during the postoperation phase and can be helpful in guiding the formulation and modification of immunotherapy treatment strategies.^472^

There has been continuous progress of nanomaterials and production technology, and nanotechnology provides great opportunities for effective control of specific immune responses and enhancement of osteosarcoma immunotherapy. However, osteosarcoma immunotherapy strategies based on nanotechnology are still in development but clearly have great potential. Therefore, multivariate diagnostic models and grading systems for osteosarcoma that consider immune components to promote accurate treatment and diagnosis of osteosarcoma should be explored. Novel methods that are more efficient than current treatment options and are also safe need to be developed, immunotherapy should be combined with other treatment modalities, and less expensive and more efficient production processes need to be pursued to achieve effective osteosarcoma treatment. Furthermore, more specific biomarkers are required for recognizing immune and nonimmune cells, identifying the interactions between the components of the immune microenvironment, exploring more suitable therapeutic targets, and integrating multidisciplinary knowledge and multitechnological support. Additionally, rational modification of the physicochemical properties of nanoparticles may be able to overcome immune escape, inhibit tumor cell proliferation and tumor progression, enhance cell targeting and drug accumulation in the tumor, and control drug release. In particular, the particle size and specific surface features of nanoparticles are special elements to be considered in the specific delivery of immunoregulators for effective targeting of the tumor microenvironment. Nanoplatforms with a size of 100 nm can be exuded from blood vessels and target tumors, spreading over a limited range in the extracellular space. However, a large number of xenograft models have confirmed that 10-100 nm nanoplatforms can readily reach and accumulate in tumor tissues after entering the blood circulatory system.^473^ Notably, nanoparticles with suitable particle size and slightly positive or negative charges have repulsive effects, which can decrease phagocytosis and clearance by the reticuloendothelial system. Consequently, the control of surface charge and steric stabilization can minimize the nonspecific interactions of nanoplatforms and prevent the loss of nanoplatforms at nontargeted sites.
